# Altered serum metabolome associated with vascular calcification developed from CKD and the critical pathways

**DOI:** 10.3389/fcvm.2023.1114528

**Published:** 2023-04-11

**Authors:** Ruyu Tan, Santao Ou, Ting Kang, Weihua Wu, Lin Xiong, Tingting Zhu, Liling Zhang

**Affiliations:** ^1^ Department of Nephrology, Affiliated Hospital of Southwest Medical University, Luzhou, China; ^2^ Department of Nephrology, The Fifth Affiliated Hospital of Southern Medical University, Guangzhou, China; ^3^ Sichuan Clinical Research Center for Nephropathy, China

**Keywords:** metabolomics, vascular calcification, chronic kidney disease, cardiovascular disease, steroid hormone biosynthesis, *in situ* synthesis of estrogens

## Abstract

**Introduction:**

Vascular calcification (VC) is more likely to be detected in the chronic kidney disease (CKD) population. The mechanism of VC development from CKD is different from that for simple VC and has always been a major research area. The aim of this study was to detect alterations in the metabolome during development of VC in CKD and to identify the critical metabolic pathways and metabolites involved in its pathogenesis.

**Methods:**

Rats in the model group were given an adenine gavage combined with a high-phosphorus diet to imitate VC in CKD. The aorta calcium content was measured and used to divide the model group into a VC group and non-vascular calcification group (non-VC group). The control group was fed a normal rat diet and given a saline gavage. Ultra-high-performance liquid chromatography-mass spectrometry (UHPLC-MS) was used to determine the altered serum metabolome in the control, VC, and non-VC groups. The identified metabolites were mapped into the Kyoto Encyclopedia of Genes and Genomes (KEGG) database (https://www.genome.jp/kegg/) for pathway and network analyses.

**Result:**

There were 14 metabolites that changed significantly in the VC group, with three metabolic pathways playing critical roles in the pathogenesis of VC in CKD: steroid hormone biosynthesis; valine, leucine and isoleucine biosynthesis; and pantothenate and CoA biosynthesis.

**Conclusion:**

Our results indicated changes in the expression of steroid sulfatase and estrogen sulfotransferase, and down-regulation of the in situ synthesis of estrogens in the VC group. In conclusion, the serum metabolome alters significantly during the pathogenesis of VC in CKD. The key pathways, metabolites, and enzymes we identified are worth further study and may become a promising therapeutic target for the treatment of VC in CKD.

## Introduction

Vascular calcification (VC) is particularly common in people with chronic kidney disease (CKD) (1), and these cases present some unique characteristics. For example, CKD primarily affects the medial layer of blood vessels, causing osteogenic differentiation of vascular smooth muscle cells (VSMCs), whereas VC developed from numerous other risk factors primarily affects the vascular intima, causing atherosclerosis (intimal calcification of blood vessels) (2). Vascular calcification caused by CKD can be divided into central artery calcification, peripheral arteriolar calcification, and valve calcification. Central artery calcification contributes to a rapid pulse wave, early pulse wave reflection, and increased cardiac afterload. These hemodynamic alterations lead to left ventricular hypertrophy, decreased coronary perfusion, and ultimately heart failure (3). Peripheral arteriolar calcification called calciphylaxis is a life-threatening complication with a high mortality rate. It is manifested as skin ulcers and necrosis caused by calcification and blockage of small arteries of the skin, accompanied by severe pain (4). Valve calcification is particularly prevalent in CKD patients. Calcific aortic valve stenosis (CAVS) can also lead to an increase in cardiac afterload. The treatment of CAVS relies on surgery as there is no evidence that the progression of CAVS can be inhibited or reduced by conservative treatment (5).VC is generally considered as an irreversible process, and therefore early diagnosis and timely treatment are necessary.

VC in CKD can be described as an imbalance of osteochondrogenic and anti-calcification factors eventually leading to osteogenic differentiation of VSMCs (6). Previous research has provided fragmented results about some of the osteochondrogenic or anti-calcification pathways and molecules. Molecules proven to be involved in VC in CKD include klotho, FGF23, matrix Gla protein (MGP), Gla-rich protein (GRP), osteoprotegerin (OPG), bone morpho-genetic protein 7 (BMP-7), and the newly found deoxycholic acid, peroxisome proliferator-activated receptor-gamma (PPARγ) (7–12).

Metabolomics is a new scientific entity after genomics and proteomics for studying life activities. In addition to genomics, metabolomics reflects the influence of environmental exposures. Proteomics has been used widely in the study of kidney diseases and has already had a substantial effect (13, 14). Metabolomics investigates small molecule metabolites (molecular weight <1,500 daltons), which are related more directly to the phenotype. The kidney is one of the main organs maintaining homeostasis of small molecule metabolites in serum (15–17), and therefore measuring alterations in the metabolome in serum during the pathological process is a good strategy for studying renal diseases.

The current study used ultra-high-performance liquid chromatography tandem mass spectrometry (UHPLC/MS) to examine the serum metabolome of rats who developed VC from CKD. We found 14 metabolites whose concentrations changed significantly in the VC group, with three metabolic pathways playing critical roles in the pathogenesis of VC in CKD. Based on these findings, we were able to predict VC in CKD using a blood test. However, the results need to be verified in CKD patients and future research is planned.

## Materials and methods

### Development of VC in rat models of CKD

All the methods were performed in accordance with the applicable international, national, and/or institutional guidelines, and was approved by the Southwest Medical University Ethics Committee (permit number 201904158). Forty-five male Sprague Dawley (SD) rats (6 weeks old and weighing 156 ± 23 g Chengdu Dossy Experimental Animal Co., Ltd., Chengdu, China) were assigned randomly to two groups: control group (*n*_0 _= 15) and model group (*n*_0 _= 30). The rats in the model group were fed a high phosphorus diet (1.8% Pi, Beijing Keao Xieli Feed Co., Ltd., Beijing, China) for 6 weeks, combined with administration of adenine (Sigma–Aldrich,LO, United States) *via* gavage given at a dose of 250 mg/kg daily for the first 4 weeks, and then once every other day for another 2 weeks. The control group was fed with saline gavage and a normal rat diet. The rats were euthanized at the end of week 6. Every rat was anesthetized with sodium pentobarbital (Amresco, WA, United States) *via* an intraperitoneal injection (45 mg/kg). After cessation of the toe pinch withdrawal response, a blood sample was collected from the abdominal aorta. Euthanasia was then conducted by CO_2_ inhalation. According to the CO_2_ replacement rate of 30% container volume/minute, and 100% CO_2_ fill at a balanced rate, the animals lost consciousness quickly. After cessation of the heartbeat, the aorta from the aortic arch to the origin of iliac artery and the kidneys was removed from the body.

Blood samples were collected from the abdominal aorta for serum chemistry tests. The aorta from the aortic arch to the origin of iliac artery, was isolated for VC evaluation and the calcium content quantified. Because the high phosphorus diet was consumed freely, rats with a body weight that deviated significantly from the average value and dead rats were excluded from the study. Using this criterion, at the end of week 6 the control group included 9 rats, while the model group included 17 rats.

### Analysis of biochemical parameters

The blood specimens were centrifuged immediately at 3,000 rpm for 10 min and the serum stored at −80°C for subsequent analysis. To evaluate kidney function, the serum creatinine level was measured using an enzymatic method (Creatinine assay kit, Siemens, MUC, Germany) and urea nitrogen by the urease method (Urea nitrogen assay kit, Siemens). Serum calcium and phosphorus levels were measured using the ion-selective electrode method (Siemens). The amount of protein excreted in a 24-hr urine sample collected 48 h before euthanasia was measured using a colorimetry method (Urine protein assay kit, Leadman, Beijing, China).

### Assessment of vascular calcification and grouping into a VC group and non-vascular calcification group (non-VC group)

The Von Kossa and MTB stains were used to evaluate the severity of VC. For each group, an aorta sample from one rat was selected randomly for Von Kossa staining (Von Kossa calcium staining kit, Genmed, Shanghai, China). The other aorta samples were frozen in liquid nitrogen and homogenized immediately for quantification of calcium content using the MTB method (Calcium determination kit, NanJing JianCheng, Nanjing, China). Rats with the highest 50% aortic calcium content were allocated to the VC group (*n* = 8), while the remainder of the rats were allocated to the non-VC group (*n* = 8).

### Non-target metabolomics study of the serum samples

#### Extraction of serum metabolites

A 100 µl aliquot of the serum samples was mixed with 400 µl of extract solution (methanol: acetonitrile = 1:1, CNW Technologies, Shanghai, China), and then pre-mixed with an internal standard (L-2-chlorophenylalanine, 2 µg/ml, Shanghai Hengbai Biotechnology Co., Ltd, Shanghai, China). The mixture was vortexed for 30 s and the samples then placed in an ice-water bath, sonicated for 10 min, followed by incubation at −40°C for 1 h. After centrifugation (10,000 rpm, 4°C, 15 min), 400 µl of the supernatant was collected in a new tube and dried in a vacuum concentrator at 37°C. The dried samples were then sonicated on ice for 10 min in 200 µl of 50% acetonitrile, followed by repeat centrifugation of the reconstituted solutions (12,000 rpm at 4°C for 15 min). After transferring 150 µl of the supernatant to a new glass vial, LC-MS analysis was carried out. Quality control samples were prepared by pooling equivalent amounts of all the supernatants (18).

#### Process of detection

The UHPLC separation was conducted using a 1,290 Infinity series UHPLC System (Agilent Technologies, MA, United States) coupled to a UPLC BEH amide column (1.7 µm, 2.1 × 100 mm; Waters, Milford, United States). The mobile phase was composed of ammonium acetate (25 mmol/L, CNW Technologies, Shanghai, China) and ammonia hydroxide (25 mmol/L, CNW Technologies), which were added to water (pH = 9.75) (A) and acetonitrile (B), respectively. The following elution gradient was used for analysis: 0–0.5 min, 95% B; 0.5–7.0 min, 95%–65% B; 7.0–8.0 min, 65%–40% B; 8.0–9.0 min, 40% B; 9.0–9.1 min, 40%–95% B, and 9.1–12.0 min, 95% B. The injection volume, auto-sampler temperature, and column temperature were 2 µl (both positive and negative modes), 4°C, and 25°C, respectively (19).

MS/MS data acquisition and analysis were conducted using the Analyst TF v1.7 (AB Sciex, MA, United States) according to a preset standard. In each cycle, a collision energy (CE) of 30 eV and cycle time of 0.56 s was used to select the densest 12 precursor ions with an intensity >100 for MS/MS. The following were set as the electrospray ionization (ESI) source conditions: curtain gas (35 psi), gas 1 (60 psi), gas 2 (60 psi), ion spray voltage floating (5,000 V in the positive mode and −4,000 V in the negative mode), declustering potential (60 V), and source temperature (600°C) (19).

### Statistical analysis

#### Pre-processing of MS raw data and metabolite identification

The mass spectrometry (MS) raw data files were converted to the extensible markup language (mzXML) format by ProteoWizard. The “XCMS” R package v3.2 was used for the following processes: peak deconvolution, alignment, and integration. Minfrac and the cut-off value were set to 0.5 and 0.3, respectively. The metabolite identification method used included m/z spectra, retention time and secondary ion fragments. The retention time (RT) of the substance was obtained by chromatographic separation, and the primary parent ion and secondary fragment ion information of the substance obtained by mass spectrometry detection. The detected mass spectrometry information was matched with the secondary mass spectrometry database built by BiotreeDB to annotate the metabolites. The recognition standard Δ rt was ±30 s and ppm (mass accuracy) ±10. In the qualitative results, the scoring value of the secondary qualitative metabolites was calculated based on the Euclidean distance and dot product algorithm (20, 21). The cut-off value of the algorithm was set as 0.3.

#### Bioinformation analysis

Data for the positive and negative ion modes were combined during the analysis. The results were expressed as mean ± SEM. Student's *t*-test was used for the univariate statistical analyses, with *p*-values <0.05 considered to be statistically significant. Multivariate statistical analysis was performed to screen the differential metabolites using orthogonal projections to latent structures discriminant analysis (OPLS-DA) and two criteria (VIP value >1 and *P*-value <0.05) (22, 23). The pathway and network analyses were conducted by mapping the differential metabolites to the KEGG database (https://www.genome.jp/kegg/) (24). All the characteristic peaks detected were used for OPLS-DA, while the volcano plots, pathway analysis, and network analysis were based on the characteristic peaks with annotations (metabolites).

## Results

### Biochemical indices of the animal models

**Figure 1:**
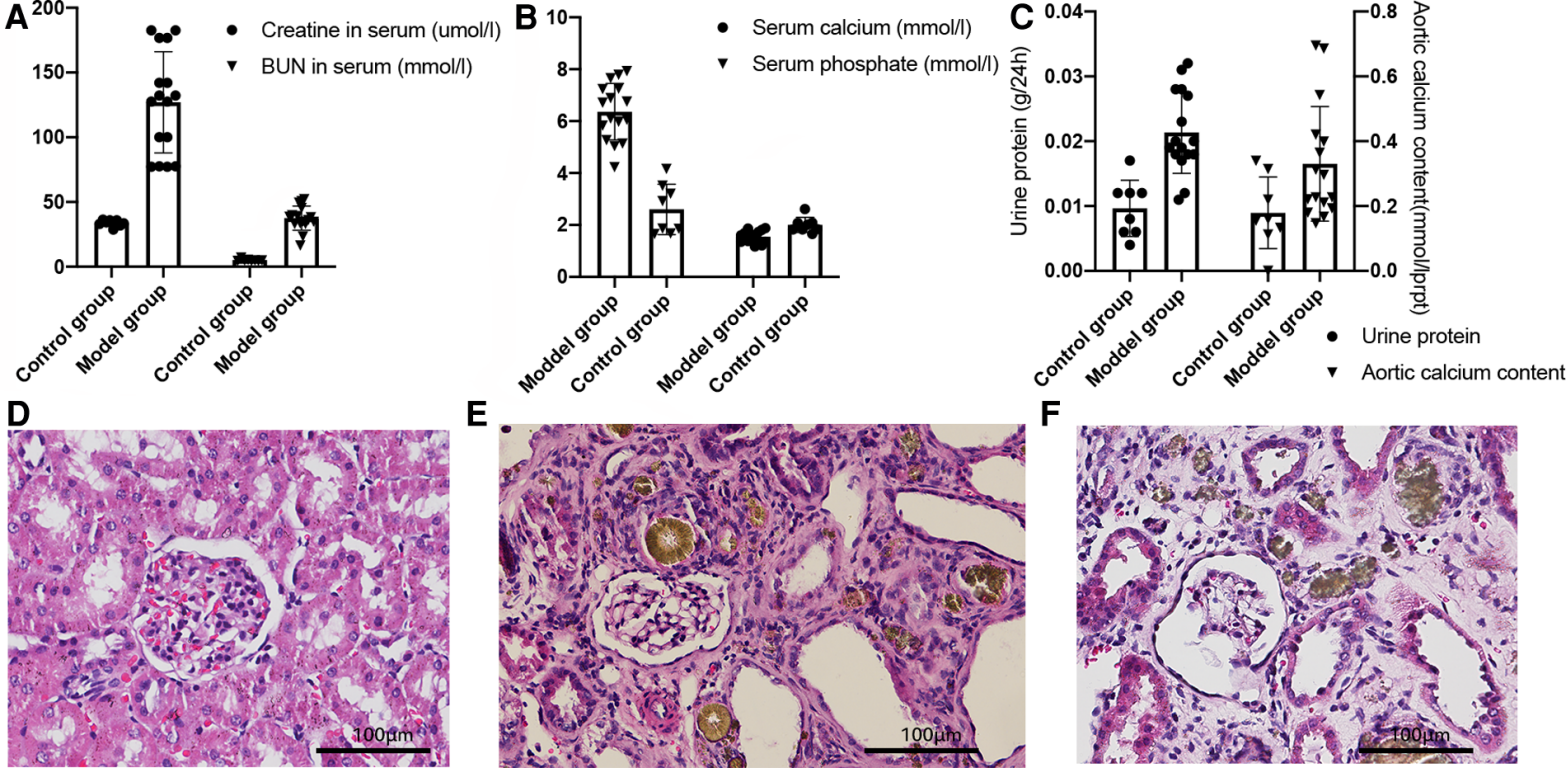
Biochemical indices of the animals analyzed by the Student's *t*-test. (**A**) Serum creatinine and blood urine nitrogen levels, *P *< 0.01. (**B**) Serum calcium and phosphate concentrations, *P *< 0.01. (**C**) 24-hour urine protein level (left, *P *< 0.01) and calcium content of the aortas (right), *P *< 0.01. (**D–F**) Kidney HE stains of the control group (**D**), non-VC group (**E**), and the VC group (**F**).

After adenine gavage administration combined with a high phosphorus diet, the model group presented with higher serum creatinine and BUN (*P *< 0.01, Figure 1A), and urinary protein levels (*P* < 0.01, Figure 1C left) than those measured in the control group. This indicated the development of renal dysfunction and successful induction of a CKD rat model. Serum calcium levels decreased while phosphorus level increased in the model group, compared to those in the control group (*P* < 0.01, Figure 1B), indicating a disorder of calcium and phosphorus metabolism.

**Figure 2:**
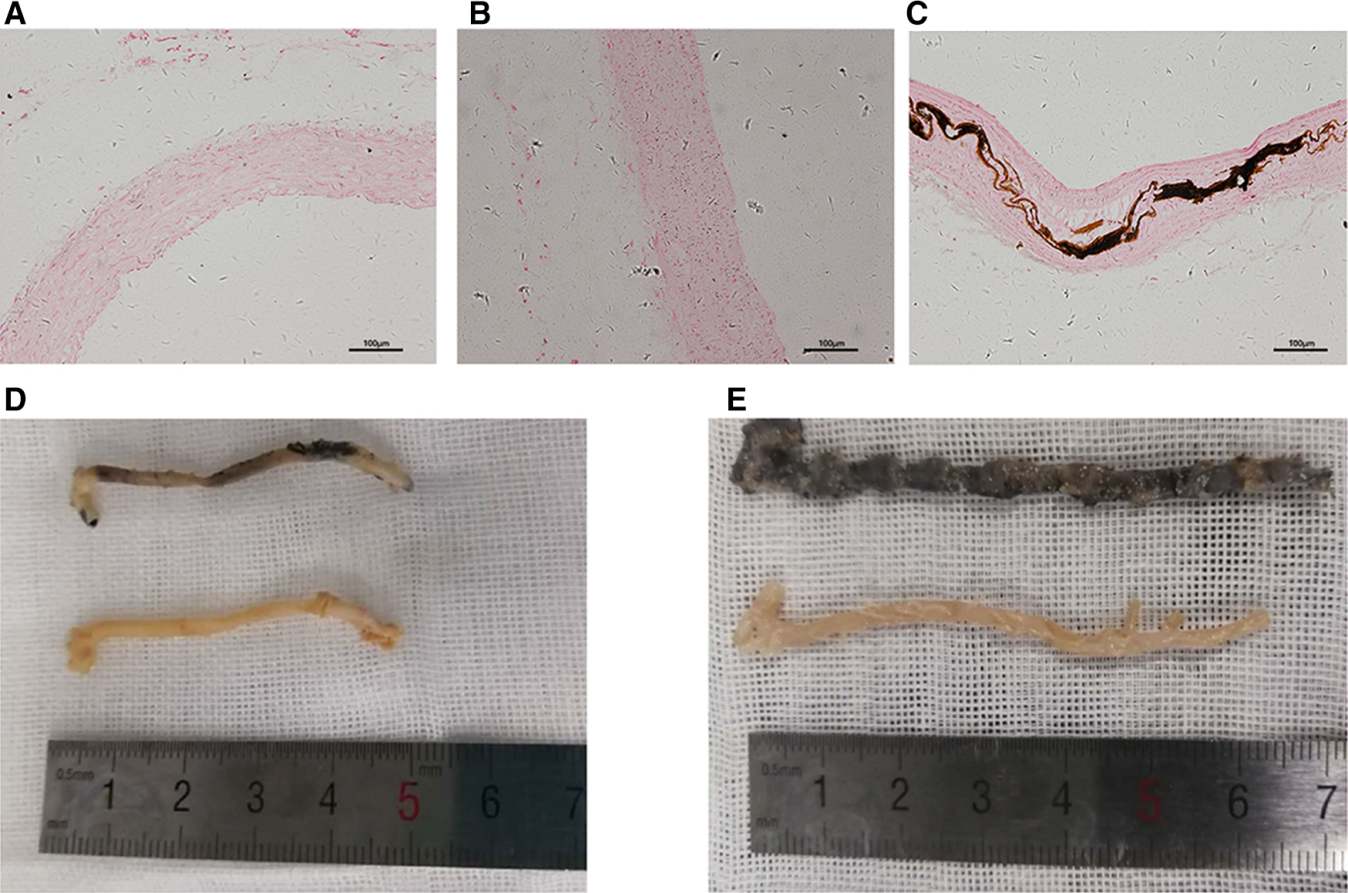
Calcification of the aorta. (**A–C**) Representative sliced aorta Von Kossa staining imaging of the control group (**A**), non-VC group (**B**) and VC group (**C**). (**D,E**) Representative aorta Von Kossa staining imaging of the control group (below) and the non-VC group (D, upper), and VC group (E, upper).

Quantification of the aortic calcium content using the methyl thymol blue (MTB) method showed that the model group had a higher calcium content compared with that in the control group (*P* < 0.01, Figure 1C right). The hematoxylin-eosin (HE) stains of the kidneys are shown in Figure 1D–F. Compared with the control group (D), the model group (E, F) showed glomerular bulging, glomerular hypertrophy, renal tubular dilatation, and inflammatory cell infiltration. Von Kossa staining of the aortas (Figure 2) showed severe VC in the VC group. As shown in Figure 2C, the VC group had calcium deposition in the medium layer of the aortic wall.

### Results of the UHPLC-MS and orthogonal projections to latent structures discriminant analysis (OPLS-DA)

**Figure 3:**
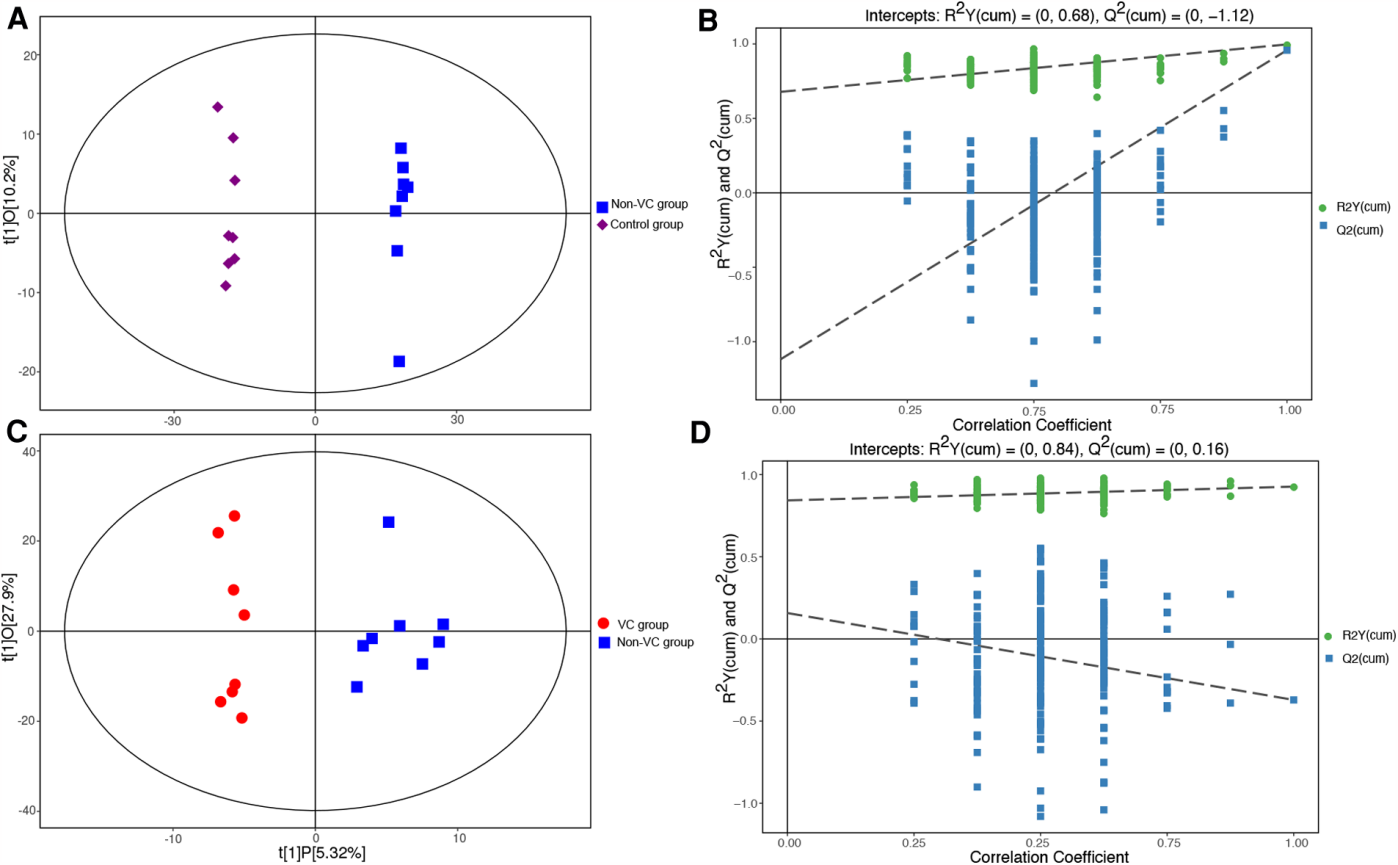
Scatter plots of the OPLS-DA scores. (**A**) Control group (purple diamonds) vs. the non-VC group (blue squares). (**B**) The permutation tests of the OPLS-DA models (control group vs. the non-VC group). (**C**) Non-VC group (blue squares) vs. the VC group (red dots). (**D**) The permutation tests of the OPLS-DA models (non-VC group vs. the VC group). The abscissa *t* [1] *P*-value represents the predicted principal component score of the first principal component, while the ordinate *t* [1] O represents the orthogonal principal component score. The scatter patterns in the different groups could be separated clearly and all were within the 95% confidence interval (Hotelling's *T*-squared ellipse). The retention of displacement of R2Y (green dots) was very close to 1. The Q2 (blue squares) of the original models was also very close to 1. The intercepts of the Q2 regression lines with the vertical axis were all <0, while the Q2 values were all lower than those of the original OPLS-DA models.

UHPLC-MS analysis identified 2,236 and 2,067 features in the positive and negative ion modes, respectively. Finally, 549 and 412 features with secondary mass spectrometry annotation were identified (metabolites) in the positive and negative ion modes (Supplementary Table S1, Initial data of UPLC-MS in the positive ion mode; Supplementary Table S2, Initial data of UPLC-MS in the negative ion mode). In the scatter plot of the scores in the OPLS-DA mode (Figure 3), the scatter patterns of different groups could be separated clearly, with all within the 95% confidence interval (Hotelling's *T*-squared ellipse). These results suggested that we could distinguish between different groups based on the serum metabolome profiles. The permutation tests of the OPLS-DA models (Figures 3B,D), verified that the original OPLS-DA models were robust, and that there was no over-fitting.

### Identification of the differential metabolites

**Figure 4:**
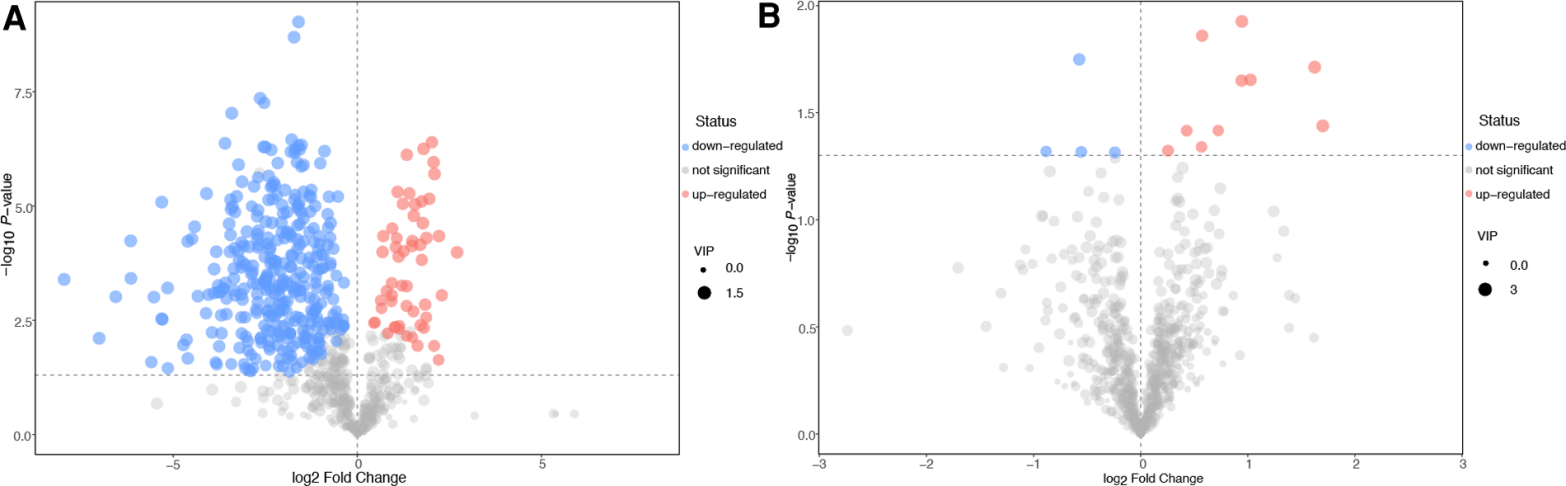
Volcano plots of the differential metabolites. Control group vs. the non-VC group (**A**). Non-VC group vs. the VC group (**B**). The abscissa represents a multiple change (base-2 logarithm) of the metabolite and the ordinate denotes the *P*-value (negative base-10 logarithm). The size of the dots indicates the VIP value in the OPLS-DA model. The upregulated metabolites are marked in red, downregulated metabolites in blue, and those with no significant difference shown as grey.

The differential metabolites (characteristic peaks with annotations) were screened using the criteria of variable importance in the projection (VIP)-value >1 and *P*-value <0.05 in the OPLS-DA models. VIP is a parameter that measures the ability to distinguish between different groups. Volcano plots (Figure 4) were prepared to show the features of the differential metabolites. In Figure 4, the sizes of the dots were consistent with the VIP value in the OPLS-DA model. The metabolites with significant upregulation are highlighted in red, those with significant downregulation are highlighted in blue, and those showing no significant difference are shown in grey. The volcano plot showed that 14 differential metabolites with significant changes in expression had been captured in the comparison between the VC and non-VC groups (Figure 4B). These metabolites included acetohydroxamic acid, alpha-ketoisovaleric acid, caprylic acid, duloxetine, estrone 3-sulfate, Gly-Lys, Ile-Phe, methyl acetoacetate, N-alpha-acetyl-L-arginine, N-acetyl-D-glucosamine, P-chlorophenylalanine, pentobarbital, tetrahydrocortisone, and trans-dehydroandrosterone. The parameters of the differential metabolites are shown in Supplementary Tables S3 and S4.

### Results of the pathway and network analyses

**Figure 5:**
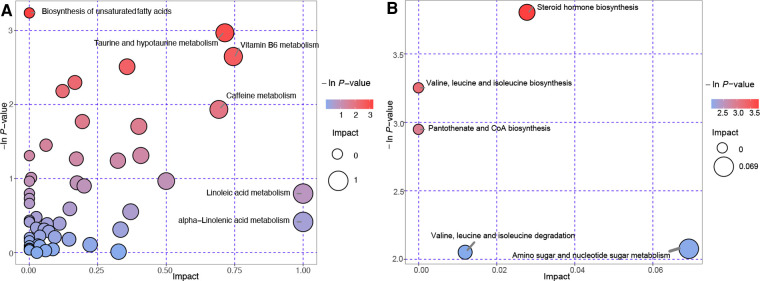
Bubble plots of the pathway analysis. Each bubble indicates a metabolic pathway. Control group vs. the non-VC group (**A**). Non-VC group vs. the VC group (**B**). The abscissa and size of the bubble represents the impact in the topological analysis of the pathway. The ordinate and color of the bubble represents the *P*-value (negative natural logarithm) in the enrichment analysis.

To identify the pathways associated with VC, the differential metabolites were mapped to the KEGG data base, followed by performance of the pathway and network analyses. The results of the pathway analysis are shown in Figure 5. Each bubble denotes a metabolic pathway, with the abscissa and the size of the bubble representing the impact of the pathway in the topological analysis. The ordinate and colour of the bubble represents the *P*-value (using negative natural logarithms) in the pathway enrichment analysis. A bubble located closer to the upper-right corner indicated that the pathway was more important. The parameters are shown in Supplementary Tables S5 and S6.

It can be seen in the comparison of the VC and non-VC groups that there are five pathways requiring attention that include steroid hormone biosynthesis, valine, leucine and isoleucine biosynthesis, pantothenate and CoA biosynthesis, valine, leucine and isoleucine degradation, and amino sugar and nucleotide sugar metabolism (Figure 5B). This shows the importance of these pathways in VC developed from CKD.

According to the interaction relationship included in the KEGG database, the metabolites, enzymes, pathways, and modules that interacted *in vivo* were connected by straight lines yielding results in the network analysis (Supplementary Figure S1). The parameters of the network analysis are shown in Supplementary Table S7. Those at the hub were considered worthy of further study. We observed that three pathways screened by the pathway analysis in Figure 5B (steroid hormone biosynthesis, KEGG number: rno00140; valine, leucine and isoleucine biosynthesis, KEGG number: rno00290; and pantothenate and CoA biosynthesis, KEGG number: rno00770) also occupied hub positions in the network analysis (Supplementary Figure S1). Estrone 3-sulfate and dehydroepiandrosterone were differential metabolites in the steroid hormone biosynthesis pathway (Supplementary Table S6). Their mass spectrum signal is enhanced in the VC group. Estrone sulfate and dehydroepiandrosterone are the most abundant circulating steroid precursors involved in *in situ* synthesis of estrogens (25, 26). Steroid sulfatase and estrogen sulfotransferase are the key enzymes that catalyse circulation estrone sulfate into biologically active estrogen. In the VC group, the accumulation of estrone 3-sulfate and dehydroepiandrosterone indicated changes in the expression of the two enzymes and down-regulation of *in situ* synthesis of estrogens.

## Discussion

By focusing on small molecules, metabolomics is perfectly suited for research on renal diseases and provides a promising tool to promote preventive strategies and maximize the potential of effective interventions (27, 28). In this study, UHPLC-MS identified a large number of features in the positive and negative ion modes. This indicated that there are abundant metabolic changes in the pathological process of CKD. Molecules previously shown to be involved in VC in CKD, including fibroblast growth factor 23, sclerostin, parathyroid hormone, bone-specific alkaline phosphatase, matrix Gla protein, osteocalcin, and osteoprotegerin, have larger molecular weight and express more specific pathophysiological processes (29–34) compared to the metabolites shown in Figure 4. Not all rats in the model group treated with adenine and high phosphorus diet showed significant calcification, which is consistent with the results of other studies (35). In fact, not all patients with end-stage renal disease are diagnosed with VC (1). The rats in the model group were consistent for age, sex, and living environment and even the genetic background was similar. This raises the question as to why these differences in the metabolome exist in the same CKD model? This may be due partly to the free intake of the high phosphorus diet in the rats. However, we excluded the rats whose body weight deviated significantly from the median value, resulting in partial elimination of this effect. Therefore, comparison of the metabolome in the non-VC and VC groups is an important approach for clarifying the pathogenesis of VC in CKD. The metabolic profile of the VC group was characterized by changes in the expression of metabolites shown in Figure 4B that included acetohydroxamic acid, alpha-ketoisovaleric acid, caprylic acid, duloxetine, estrone 3-sulfate, Gly-Lys, Ile-Phe, methyl acetoacetate, N-alpha-acetyl-L-arginine, N-acetyl-D-glucosamine, P-chlorophenylalanine, pentobarbital, tetrahydrocortisone, and trans-dehydroandrosteron. These metabolites may be markers of VC developed from CKD.

The metabolites identified were projected into the KEGG database to identify the relevant pathways. The result of the pathway analysis emphasized “steroid hormone biosynthesis” (Figure 5B). Network analysis also showed that “steroid hormone biosynthesis” occupied a central position in the pathogenesis of VC developed from CKD. Estrone 3-sulfate and dehydroepiandrosterone were differential metabolites in the steroid hormone biosynthesis pathway in the VC group (Supplementary Table S6). Estrone sulfate and dehydroepiandrosterone are the most abundant circulating steroid precursors involved in *in situ* synthesis of estrogens (25, 26). It is considered that estrogen binding to the estrogen receptor on VSMCs plays an anti-atherosclerotic role (36). Our results indicated down-regulation of *in situ* synthesis of estrogens in the VC group, which may weaken the anti-atherosclerotic effect of estrogen on blood vessels. Steroid sulfatase and estrogen sulfotransferase are two enzymes that catalyse estrone sulfate into biologically active estrogen. Shchelkunova et al. used quantitative PCR to analyse the changes in gene expression in human aortal intima during atherogenesis and showed changes in the expression of these two enzymes (37). Their other research also showed lower levels of steroid sulfatase and higher levels of estrogen sulfotransferase in blood vessel fragments with atherosclerotic lesions (38), indicating down-regulation of *in situ* synthesis of estrogens had occurred in the calcified blood vessels. Rose et al. performed immunohistochemical staining on the coronary arteries of 55 women. They showed ER was expressed in all artery wall layers, but most avidly in the media and colocalized with VSMCs (39). In recent years, many scholars have tried to clarify the mechanism by which estrogen attenuates VC, but were not able to provide a direct link between the estrogen-estrogen receptor pathway in VSMCs and VC in CKD. The findings of our experiment indicate that malfunction of *in situ* synthesis of estrogens are involved in VC developed from CKD and provide metabolic support for the protective effect of estrogen on VC in CKD.

Alpha-ketoisovaleric acid is a differential metabolite in three pathways: valine, leucine, and isoleucine biosynthesis, pantothenate and CoA biosynthesis, and valine, leucine, and isoleucine degradation (Figure 5B). Supplementary Table S5 shows that alpha-ketoisovaleric acid was significantly up-regulated in the VC group. Alpha-ketoisovaleric acid is the keto analogue of valine, and has only two possible metabolic endpoints: transamination to valine or oxidative decarboxylation to isobutyric acid (40). A research study on stem cell-based therapies for atherosclerosis (calcification of the vascular intima) compared the metabolic profiles of bone marrow-derived mesenchymal stem cells and adipose-tissue derived mesenchymal stem cells (ADSCs) using liquid chromatography quadrupole time-of-flight mass spectrometry. The results showed that alpha-ketoisovaleric acid is one of the metabolites responsible for the potential differences in the metabolic characteristics of BMSCs and ADSCs (41). Currently, alpha-ketoisovaleric acid together with other keto analogues of essential amino acids are used widely to delay the progression of renal failure in patients with CKD. However, there is a lack of studies that have investigated the relationship between the metabolism of alpha-ketoisovaleric acid and vascular calcification in CKD. Wu et al. observed an alteration in pantothenate and CoA biosynthesis in patients with coronary heart disease (42), while Zhao et al. reported that astaxanthin promotes differentiation of mesenchymal stem cells (MSCs) into osteoblasts by influencing pantothenate and CoA biosynthesis and two other pathways (43). These reports together with the results of our pathway analysis suggest that pantothenate and CoA biosynthesis should be areas of interest in VC research. The next two pathways in Figure 5B (valine, leucine, and isoleucine biosynthesis and valine, leucine and isoleucine degradation) were involved in the metabolism of branched-chain amino acids (BCAA). Zuzana et al. considered that dysmetabolism of BCAA may promote VC by influencing endothelial cell remodeling (44), although there is lack of evidence for this possibility.

We also compared the non-VC group with the control group to identify the altered metabolic profile in CKD. The volcano plots showed that numerous metabolites changed during CKD pathogenesis (Figure 4A), indicating that CKD is a disease with obvious metabolic changes. Pathway analysis also showed that several pathways were involved, including biosynthesis of unsaturated fatty acids, taurine and hypotaurine metabolism, vitamin B6 metabolism, caffeine metabolism, linoleic acid metabolism, and alpha-linolenic acid metabolism. These findings contribute to our further understanding of CKD metabolism.

## Conclusions

In this study, UHPLC-MS was used to determine the altered metabolic spectrum of VC developed from CKD. OPLS-DA identified 14 differential metabolites in the VC group compared to those observed in the non-VC group. These metabolites maybe the biomarkers of VC developed from CKD. Five metabolic pathways were screened out in the pathway analysis. Of these pathways, steroid hormone biosynthesis, valine, leucine and isoleucine biosynthesis, pantothenate and CoA biosynthesis also occupied hub positions in the network analysis. The results of the pathway and network analyses suggested that the steroid hormone biosynthesis pathway may be an entry point for a therapeutic strategy for treating VC in CKD. BCAAs metabolic changes may also be involved in the process of VC in CKD. Comparison between the control and non-VC groups indicated that CKD is a disease with marked metabolic changes. UHPLC-MS is a highly sensitive technique, while animal experiments with well controlled additional variables are necessary to study these metabolic changes. Clinical trials are also needed to verify whether the 14 differential metabolites have the potential to assist with the diagnosis of VC in CKD, while the therapeutic value of the pathways requires further investigation.

## Data Availability

The raw data supporting the conclusions of this article will be made available by the authors, without undue reservation.
