# Supplementary figures and images for: Altered serum metabolome associated with vascular calcification developed from CKD and the critical pathways
Source: Front Cardiovasc Med. 2023 Apr 11;10:1114528. doi: 10.3389/fcvm.2023.1114528 (PMC10126378; doi:10.3389/fcvm.2023.1114528)

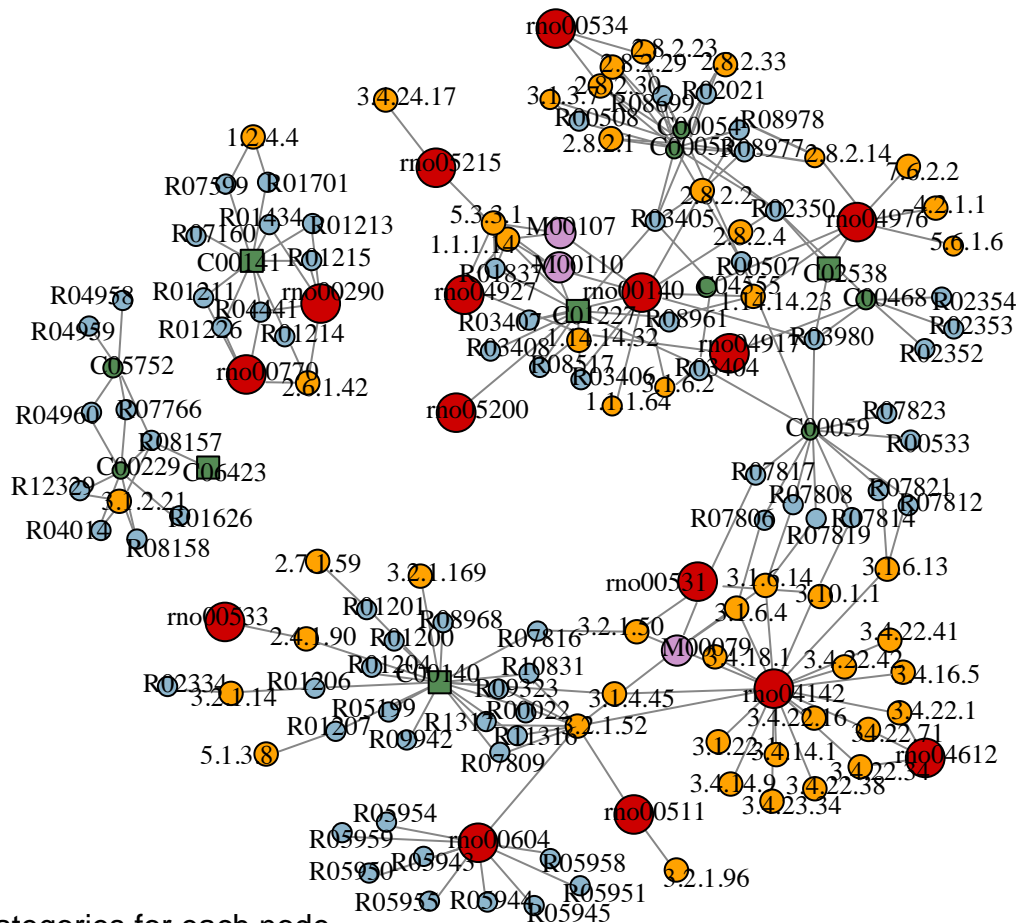

Supplement: Supplementary file 8 [file Datasheet1.pdf]
